# Implementing Alcohol Misuse SBIRT in a National Cohort of Pediatric Trauma Centers—a type III hybrid effectiveness-implementation trial

**DOI:** 10.1186/s13012-018-0725-x

**Published:** 2018-02-22

**Authors:** Michael J. Mello, Sara J. Becker, Julie Bromberg, Janette Baird, Mark R. Zonfrillo, Anthony Spirito

**Affiliations:** 1 Department of Emergency Medicine, Alpert Medical School of Brown University, Providence, RI USA; 2 Department of Health Services, Policy and Practice, Brown University School of Public Health, Providence, RI USA; 3 Injury Prevention Center of Rhode Island Hospital-Hasbro Children’s Hospital, Providence, RI USA; 4 Department of Behavorial and Social Sciences, Brown University School of Public Health, Providence, RI USA; 5 Department of Pediatrics, Alpert Medical School of Brown University, Providence, RI USA; 6 Department of Psychiatry and Human Behavior, Albert Medical School of Brown University, Providence, RI USA

**Keywords:** SBIRT implementation, Alcohol, Drugs, Pediatrics, Trauma center

## Abstract

**Background:**

The American College of Surgeons mandates universal screening for alcohol misuse and delivery of an intervention for those screening positive as a requirement for certification as a level 1 trauma center. Though this requirement has been mandated for over a decade, its implementation has been challenging. Our research team completed an implementation study supporting seven pediatric trauma centers’ compliance with the requirement by developing and implementing an institutional alcohol Screening, Brief Intervention and Referral to Treatment (SBIRT) policy for adolescent trauma patients. A mixed-methods approach indicated that SBIRT adoption rates increased at all sites; however, providers’ fidelity to the SBIRT intervention was variable, and providers reported a number of barriers to SBIRT implementation. The goal of this study is to conduct a fully powered type III hybrid effectiveness-implementation trial to test the effectiveness of a comprehensive implementation strategy in increasing the implementation of SBIRT for alcohol and other drug use (AOD) in pediatric trauma centers.

**Methods:**

Our implementation strategy is based on the Science to Service Laboratory (SSL), an approach developed by the SAMHSA-funded Addiction Technology Transfer Centers that consists of three core elements (i.e., didactic training + performance feedback + leadership coaching). Utilizing a stepped wedge design, a national cohort of 10 pediatric trauma centers will receive the SSL implementation strategy. At six distinct time points, each of the 10 sites will provide data from 30 electronic medical records (*n* = 1800 in total). A subset of adolescents will also report on fidelity of intervention delivery and linkage to care (i.e., continued AOD discussion and/or treatment with a primary care provider) 1 month after hospital discharge. In addition, nurses, social workers, and leaders will report on organizational readiness for implementation at four distinct time points.

**Discussion:**

This protocol proposes a unique opportunity to examine whether a comprehensive implementation strategy can improve the fidelity of SBIRT delivery across a national cohort of pediatric trauma centers. With injured adolescents, this could optimize the detection and intervention of AOD use and improve adolescent health.

**Trial registration:**

Clinicaltrials.gov NCT03297060.

## Background

Alcohol use increases throughout adolescence with greater usage rates and escalation occurring in older adolescents. The proportion of youth who report past month alcohol use increases almost fourfold from 9.7% of 8th graders to 35.3% of 12th graders [1]. Youth who reported initiating drinking before the legal age were significantly more likely to have been injured while drinking and/or to have unintentionally injured themselves or others relative to same age peers [2, 3].

About one third of hospitalized adolescent trauma patients screen positive for alcohol misuse [4]. Due to the relationship between adolescent drinking and injury, pediatric trauma centers represent an opportune setting to intervene with youth at risk of alcohol use disorders. Since 2006, the American College of Surgeons Committee on Trauma (ACS-COT) has recommended that all trauma centers have the capacity to identify patients who are problem drinkers and has mandated that level 1 trauma centers have a mechanism to provide these patients with a brief intervention (BI) [5]. Since then, the Screening, Brief Intervention and Referral to Treatment (SBIRT) model has gained traction and become the most widely endorsed public health approach to improve the detection of and intervention for alcohol misuse in acute care settings. The goal of the SBIRT model is to use universal screening (S) to identify those individuals at risk of alcohol and other drug (AOD) use disorders, administer an appropriate brief intervention (BI), and initiate referral to treatment (RT) [6]. Although pediatric trauma centers are supportive of SBIRT [8], reported screening rates for AOD use are low, ranging from 26 to 50% [9–12] demonstrating a need for an improved implementation strategy.

Additionally, many trauma patients are discharged on pain medications to address pain control from their injuries. US pediatric trauma centers are faced with the challenge of appropriately addressing pain control and preventing opioid misuse in the current environment of an opioid misuse epidemic [13–16]. The SBIRT model offers an opportunity for incorporating counseling about prescription pain medication usage into the BI for those at high risk for misuse.

In a prior study, we assisted seven pediatric trauma centers in developing and implementing an SBIRT protocol to comply with the ACS mandate [8]. Our approach consisted of three components: (a) didactic training (i.e., upfront workshop and annual in-person meetings), (b) performance feedback (i.e., ongoing electronic medical record review and feedback to site leaders), and (c) staff coaching (i.e., identification of site SBIRT champions, consultation to front-line staff, and monthly calls with SBIRT champions). At the end of the study, screening rates increased from 11 to 73% of eligible patients, with 90% of those who screened positive receiving an indicated BI. While our results were encouraging, the original study did not measure fidelity to the protocol (i.e., whether SBIRT was administered correctly), assess putative mediators of implementation effectiveness, or track the RT component of the SBIRT model. In addition, feedback from staff indicated two barriers to sustained implementation: lack of integration into the electronic medical record and lack of customized training for nurses, social workers, and leaders, all of whom had different training needs. The current study addresses these barriers via a comprehensive implementation strategy called the Science to Service Laboratory (SSL), which we enhance by integrating the intervention into the electronic health record (EHR) and developing separate training tracks for nurses, social workers, and organizational leaders. We deliver the SSL in partnership with the New England Addiction Technology Transfer Center (ATTC), a federally funded addiction training center, to leverage publicly available training materials and optimize scalability.

The goal of this study, Implementing Alcohol Misuse Screening, Brief Intervention and Referral to Treatment (IAMSBIRT), is to conduct a fully powered type III hybrid effectiveness-implementation [17] trial to test the effectiveness of a comprehensive implementation strategy in promoting fidelity to SBIRT delivery in pediatric trauma centers. Our primary study aim is to evaluate the effectiveness of the SSL implementation strategy in increasing fidelity of SBIRT delivery at pediatric trauma centers, relative to usual implementation. We hypothesize that after receiving the SSL, there will be an increase of 20% in the proportion of admitted injured adolescents receiving a validated AOD screening, as well as each element of the SBIRT protocol indicated based on the screening: (a) brief AOD intervention and (b) referral to appropriate care post-discharge (i.e., continued AOD discussion/treatment with a health care provider). We have secondary aims of evaluating: (a) whether readiness for organizational change mediates the influence of the SSL implementation strategy on implementation effectiveness (i.e., fidelity of SBIRT delivery) and (b) the effect of the SSL implementation strategy on improving patient linkage to appropriate care following discharge from pediatric trauma centers (i.e., continued AOD discussion with primary care provider and/or AOD treatment). We also have an exploratory aim examining the integration of counseling regarding the use of prescription pain relievers into SBIRT delivery with injured adolescent patients who screen positive for AOD use.

## Methods

### Study design

**Fig. 1:**
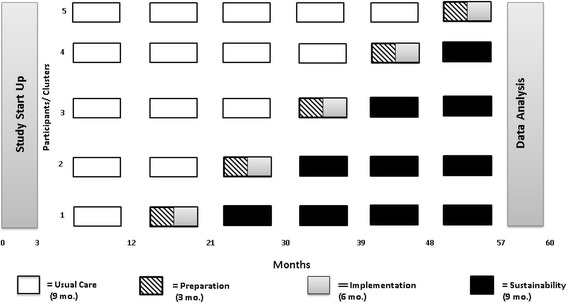
Implementing Alcohol Misuse Screening, Brief Intervention and Referral to Treatment (IAMSBIRT) stepped wedge design and timeline. Usual care is denoted as a solid blank square and signifies a time period of 9 months. Preparation stage is denoted as a box with diagonal lines and signifies a time period of 3 months. Implementation stage is denoted as a solid gray box and signifies a time period of 6 months. The sustainability stage is denoted as a solid black box and signifies a time period of 9 months

Utilizing a unidirectional crossover stepped wedge design, a national cohort of ten pediatric trauma centers will receive the SSL implementation strategy. A stepped wedge design is a sequential roll-out of an intervention (i.e., the SSL implementation model) over a number of discrete time points. In the proposed study, we will randomly assign ten pediatric trauma centers to one of five cohorts (two sites in each cohort step) that will determine when they crossover from control to SSL. Computer-generated random numbers will be used to determine the randomized order of the wedge assignments. The research staff member responsible for randomization will not share the sequence order with the sites or the research team. Data are collected over six assessment periods that are 9 months in duration. During the first assessment period (T0), none of the ten participating pediatric trauma centers receive the SSL. During the second assessment period, the first randomly selected cohort of two pediatric trauma centers completes the SSL preparation and implementation phases. Over the third assessment period, the first cohort shifts to the sustainment phase, the second cohort receives the SSL preparation and implementation phases, and the remaining cohorts stay in the control condition. This crossover pattern continues until year 5 when all the trauma centers have progressed form control condition through to active implementation phase (see Fig. 1).

### SBIRT intervention model

All pediatric trauma centers will implement the following SBIRT protocol for pediatric trauma patients to identify substance use/misuse and to provide BI and referral to treatment, as appropriate, once their site is randomized to the SSL implementation model.

#### Screening (S)

Injured patients age 12–17 years admitted to the hospital will have (1) their blood alcohol level (BAC) and urine drug screen (UDS) collected during their initial trauma evaluation and (2) receive the Screening to Brief Intervention Tool (S2BI [18]; embedded in the EHR) during their initial evaluation by inpatient nursing staff. S2BI is a newly validated screening for youth which assesses past year use of eight substances, including alcohol. This screening tool is recommended in the American Academy of Pediatrics’ (AAP) most recent policy statement and has a reported sensitivity of 90% and specificity of 94% detecting any AOD disorder for adolescents who report more than monthly use [19]. A design template of the S2BI will be provided to each institution to ease institutional EHR integration. This assessment will be administered and scored as recommended [20]. Specifically, all patients will be asked three screening questions about their past year use of alcohol, tobacco, and marijuana by a nurse. If the adolescent responds “never” to all three questions, the nurse will provide positive reinforcement. Those reporting past year use on one or more of the screening questions will be asked four additional questions on past year drug use. All adolescents reporting any past year AOD use will receive a social work consult.

#### Risk algorithm

The social work consult will begin with review of the S2BI results to triage adolescents into three categories: (a) substance use (past use, once, or twice) but not substance use disorder (SUD), (b) mild-moderate SUD (past use monthly), and (c) moderate-severe SUD (past use weekly or more often). Those adolescents in the substance use but no SUD group will receive brief advice using a standard script: the social worker will recommend that the adolescent quit using because substance use can get in the way of their goals.

#### Brief intervention (BI)

All adolescents who report AOD use monthly or more often will receive a brief intervention in motivational interviewing (MI) style (i.e., brief motivational interview; BMI). A BMI is a short, structured conversation based on the principles of MI in which the clinician explores problems, recognizes ambivalence, and listens for “change talk” from the teenager [21]. The BMI follows a structured format to increase speed and efficiency. The social worker will ask adolescents what specific changes related to their AOD use they are willing to implement and will guide adolescents in discussion of a concrete change plan. Adolescents meeting with the social worker for brief advice or a BI will also receive counseling about safer use of prescription pain reliever medication after discharge and safe medication disposal.

#### Referral to treatment (RT)

In the traditional SBIRT model, RT is defined narrowly as referral to AOD services for those with moderate to severe SUD. In this study, we define the RT phase more broadly as linkage to follow-up discussions about AOD with a health care provider. Consistent with the current AAP guidelines [7], the social worker will recommend that all adolescents with a history of AOD use receive linkage to the primary care provider (i.e., the adolescent’s “medical home”) for a follow-up discussion. Participants with moderate to severe SUD will also receive referral to AOD treatment.

### Study participants

We define our participants at three levels: organization (pediatric trauma centers), adolescent trauma patients, and staff (nurses, social workers, and institutional leaders).

We selected the participating ten pediatric trauma centers based on the following criteria: (a) certified as a level one pediatric trauma center, (b) did not participate in our prior research study on this topic, and (c) self-identified as willing to receive SBIRT assistance on an Injury Free Coalition for Kids survey [22]. Participating sites include Arkansas Children’s Hospital, Boston Children’s Hospital, Dell Children’s Medical Center, Harborview Medical Center, Hasbro Children’s Hospital, Hennepin County Medical Center, Johns Hopkins University–Bloomberg Children’s Center, Yale-New Haven Children’s Hospital, Intermountain Primary Children’s Medical Center, and UMass Memorial Children’s Medical Center.

The primary source of patient data extraction is the EHR. Within each 9-month wedge period, we will send sites a randomly selected date and have them extract de-identified data from the next 30 admissions occurring after this date. By the end of the study, each site will be responsible for abstracting 180 (30 medical records × 6 “wedges”) medical records, resulting in 1800 medical records across sites. An explicit mechanism for EHR data abstraction will be utilized across all sites.

Data for secondary aims will be collected directly from patients and providers. Following a similar defined protocol across all sites to identify adolescent patients, each site will recruit and consent one adolescent per month during their hospital stay who screened positive for alcohol use to further assess patient-level data at a follow-up assessment 30 days after their hospital discharge. Data collected will include (a) adolescent report of staff delivery of SBIRT components during their time at the pediatric trauma center and (b) adolescent report of follow-up conversations with the primary care provider following discharge from the pediatric trauma center. A total of 540 adolescents (10 sites × 9 adolescents per wedge period × 6 wedges) will be recruited for the post-discharge patient assessments that will be conducted by telephone. To optimize ecological validity, exclusion criteria for these post-discharge assessments will be kept to a minimum. Eligible adolescents must meet these criteria: (a) between the ages of 12–17 years; (b) admitted to a participating trauma service for an injury; (c) screened positive for AOD use based on biologic testing or self-report on the alcohol screening tool (for example: S2BI); (d) youth, in the opinion of the clinical staff, is medically stable to participate in study enrollment; (e) youth accompanied by a parent of legal authorized representative (LAR); (f) youth and parent able to speak English or Spanish; (f) youth able to provide written assent and parent able to provide written consent; and (g) youth has telephone or email access. Exclusion criteria include prisoner or in police custody and those who were admitted due to suicide attempt and any acute conditions that would preclude provision of informed consent (i.e., acute psychosis, altered mental status, cognitive impairment).

All nurses and social workers caring for patients admitted to the pediatric trauma service at each center will receive SBIRT training. We will also recruit three leaders per site: one will be responsible for overseeing nursing, one will be responsible for overseeing social workers, and one will be responsible for general oversight. The 30 leaders (10 sites × 3 leaders per site) will participate in the SSL leadership track (described below). In addition, staff in all three tracks—nurses, social workers, and leaders—will be asked to complete a series of anonymous brief online assessments. The assessments will contain measures of putative mediators and predictors of SBIRT implementation effectiveness and will be administered at four different time points—baseline, 1 month before their wedge’s preparation phase, immediately after their implementation phase, and at the end of the study period.

### Implementation condition

**Fig. 2:**
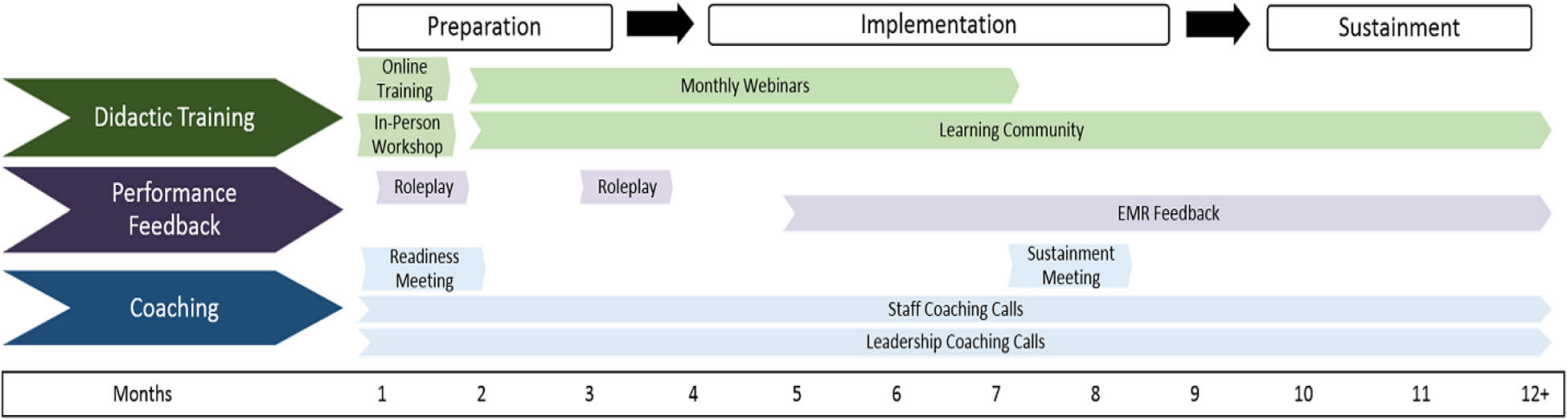
Specific elements of the Science to Service Laboratory (SSL) implementation strategy

The SSL consists of three components: didactic training + performance feedback + external coaching. To promote scalability, the SSL training materials will rely heavily on free, publicly available materials from the ATTC Network. Expanding upon prior research conceptualizing implementation as a “process” that occurs over multiple stages [23, 24], we have divided implementation activities into three key phases: preparation, implementation, and sustainability. The 9-month SSL protocol (i.e., didactic training + performance feedback + coaching) encompasses both preparation and implementation activities and then it moves into the sustainability phase. An overview of specific SSL activities contained within each phase is depicted in Fig. 2.

#### Preparation phase

The SSL model begins with preparation activities that occur before the first patient is engaged. The objectives of this phase are to promote organizational readiness for implementation and consist of didactic training for all three tracks, formal performance feedback for social workers based on role-plays, and an in-person meeting between center leadership and the external research team. Pediatric trauma centers will also receive targeted support from a medical clinical informatics consultant focused on integrating SBIRT into the EHR. By the end of this 3-month stage, organizational leaders are expected to have committed resources (EHR integration and development of quality improvement (QI) procedures) to engage in implementation.

##### Didactic training: online workshop

Each track will be required to complete an online workshop and will have access to other optional workshops; continuing education credits will be provided. We will provide a 30-min online training for nurses specifically focused on screening and a 90-min training for social workers specifically focused on BI/RT. Leaders will be required to complete the full training. Content of the online workshops covers rationale for implementing SBIRT with adolescents (all), rationale for universal screening and age-appropriate validated tools (nurses, leaders), rationale for brief intervention and age-appropriate intervention elements (social workers, leaders), rationale for providing education on prescription pain reliever misuse (social workers, leaders), and rationale for post-discharge referrals and coordination with the primary care doctor (social workers, leaders).

##### Didactic training: in-person workshop

Following completion of the online courses, members of the research team and a regional ATTC SBIRT coach will make a 1-day visit to each site. As part of this visit, there will be a half-day “hands on” training workshop focused on the practice of SBIRT skills. This workshop will be required for organizational leaders and social workers and encouraged for nurses. Topics covered in the workshop will include interpreting the results of the S2BI, practicing BI (including the provision of opioid pain medication education) and RT, documenting the delivery of BI and RT in the EHR, and reviewing of training and supervision plans. Training strategies used during the workshop will include case examples, videotape vignettes, and active role-plays. Workshop content will be standardized across the ten sites and based on the 2015 Massachusetts Adolescent SBIRT Toolkit for Providers [20]. The ATTC national registry of certified SBIRT trainers from surrounding areas to the participating trauma center will serve as workshop leaders.

##### Didactic training: monthly webinars

Separate monthly webinar series will be organized for site leaders, nurses, and social workers. These webinars start during the preparation phase and continue into the next implementation phase. Each track will contain three to six monthly webinars, which will be selected from the ATTC Network’s online compendium of SBIRT resources. Site leaders will be required to attend six monthly webinars focused on SBIRT implementation and sustainment. The first leadership webinar will focus on how to integrate SBIRT processes into the EHR. Participation in monthly webinars by nursing and social workers will be optional (with continuing education credits offered), and we will track completion as an indicator of staff engagement. Nurses will be offered three webinars on screening (e.g., SBIRT guide: an introduction to screening; use of the S2BI; integrating screening in the EHR), whereas social workers will be offered six webinars on BI and RT (e.g., principles of motivational interviewing, normative feedback about alcohol and opioid misuse, reimbursement and coding for BI, referral to treatment for alcohol and opioid misuse, integrating SBIRT in the EHR).

##### Didactic training: learning community

For each of the five cohorts in the stepped wedge trial, separate learning communities will be organized for nurses, social workers, and leaders. Specifically, each learning community will be offered the same monthly webinar series (described above) and will be subscribed to an electronic mailing list via which participants can freely ask questions and exchange information. The webinar series will be offered using a platform that enables live interactions; immediately after each webinar, attendees will be presented with a case example and members will have the opportunity to engage in real-time discussion about how to address the case. The three designated leaders from each site will be subscribed to the leader track electronic mailing list, and as well as the electronic mailing lists for the nursing and social worker tracks to ensure awareness of implementation progress. The electronic mailing lists will be monitored by the research team throughout the preparation and implementation stages to ensure that appropriate follow-up activities (i.e. providing accurate information on the electronic mailing lists, retraining staff) occur. Participation in the learning community will be measured as an indicator of staff engagement.

##### Performance feedback: role-plays

Before the end of the 3-month preparation phase, social workers and leaders will receive performance feedback on two practice cases. At the end of the half-day workshop, social workers and leaders will role-play BI sessions using standardized patient scripts. Live feedback on fidelity will be provided immediately after the role-play. Those who do not demonstrate satisfactory fidelity during the live role-play will be given specific corrective feedback. Following the workshop, social workers will have 3 months to submit an audio recording of a role played BI session with the designated social worker leader. Role-plays will again use a standardized patient script and will be rated by research staff for fidelity. Social workers will receive performance feedback on their fidelity to the risk algorithm.

##### External leadership coaching

Throughout this 3-month phase, the SSL external leadership coach will hold monthly site-specific coaching calls for track leaders to discuss the goals of SBIRT implementation, address any issues that have come up related to staff engagement in training, and discuss ways to increase organizational preparedness for SBIRT implementation. Near the end of this phase, the research team will work with three designated organizational leaders from each pediatric trauma center to conduct an in-person planning meeting to review results of the scales measuring putative mediators and predictors (i.e., ORIC, SBIRT knowledge). These data will be used to identify primary barriers to SBIRT implementation and develop a plan to address them. Plans to ensure integration of SBIRT into the EHR and to develop continuous QI procedures assessing SBIRT delivery will also be discussed.

#### Implementation phase

During this 6-month stage, trained staff will begin active SBIRT implementation.

##### Didactic training: webinars and learning community

Throughout this phase nurses, social workers, and leaders will continue to be offered monthly webinars and will continue to have access to the learning community described above.

##### Performance feedback

Throughout the implementation phase, designated leaders are responsible for generating performance feedback data based on EHR data as part of their QI protocol. For the nursing track, the designated nursing leader will generate data on each nurse’s screening fidelity (e.g., was the S2BI administered, were responses documented, were biologics obtained) and will be responsible for sharing this performance feedback with each nurse. The social worker track leader will similarly generate data about each social worker’s BI/RT fidelity (e.g., was BI or brief advice administered, was opioid education given, was referral to treatment documented, was disposition appropriate given results of the screening) and will be responsible for sharing performance feedback with social workers. The site leader will be responsible for ensuring that the nursing and social worker track leaders are providing performance feedback. The site leader will provide the research team with QI metrics for both nursing and social workers each month during implementation and then at 9-month intervals after that for the duration of the grant. At the end of the implementation phase, each pediatric trauma center will be provided with site-specific performance feedback on the fidelity of SBIRT delivery.

##### External leadership coaching

Near the end of this phase, the SSL external leadership coach will meet with each pediatric trauma center for a face-to-face sustainability planning meeting. This meeting will focus on creating concrete action plans for the continuation of SBIRT implementation after the removal of active support.

#### Sustainability phase

During this phase, the New England ATTC will continue to be available for support such as offering of new webinars, access to learning community electronic mailing lists, and access to conference lines for monthly coaching calls. However, active provision of support from the external leadership coach will cease. We will continue to measure staff participation in implementation activities and will continue to monitor EHR data, to provide an ecologically valid indicator of organizational sustainment.

### Measuring study aims

#### Primary aim—implementation effectiveness: EHR Trauma Data Collection Tool

The EHR Trauma Data Collection Tool will be used by the study sites to record the extracted SBIRT appropriate data on the admitted adolescent patient from the EHR. Data points to be collected include results of laboratory alcohol screening instrument, results of alcohol screening questionnaire, referred for brief advice (Y/N), received brief advice (Y/N), referred for a brief intervention (Y/N), received brief intervention (Y/N), referred for treatment (Y/N), type of referral to treatment made (if applicable), and referral back to medical home (Y/N). Data will be de-identified and electronically transferred to the research team at for appropriate coding. Because the EHR will capture the S2BI results, we will be able to determine not only if the pediatric trauma center staff delivered the specific SBIRT components (i.e., adoption of SBIRT) but will also be to determine if the S2BI risk algorithm was followed correctly (i.e., fidelity of SBIRT). We will calculate site-specific rates for % of eligible patients screened with a validated tool, % of patients who received an *indicated* brief intervention, and % of patients who received an *indicated* referral for continued discussion about AOD misuse (with either their PCP/pediatrician/medical home or an outpatient treatment provider). To ensure the reliability of EHR data, we will require each site to do re-extraction of 10% of the data at each site. We will assess this data for confirmation of the validity of the data extraction methods and quantify the concordance by calculating the kappa statistic.

#### Secondary aim—mediator of implementation effectiveness: organizational readiness for implementing change (ORIC)

This 12-item scale will be implemented to all participating staff at four time-points. Staff responses will be used to create an organizational-level measure of each pediatric trauma center’s shared perceptions regarding the extent to which their center has the level of skill, training, time, and resources necessary to implement the SBIRT protocol. This measure has been adapted for the proposed study based on the implementation readiness measure previously developed [25].

#### Secondary aim—measure of patient effectiveness: Adolescent Perception of SBIRT Services Survey

During the 30-day post-discharge assessment, we will utilize the Adolescent Perception of SBIRT Services Survey which was developed by the research team based on the screening questions from S2BI to evaluate patient experience of SBIRT services and patient linkage to care after BI. This survey consists of 12 items with yes/no response options asking the adolescent about the specific services received (e.g., were they asked about AOD use; did a social worker talk to them about their AOD use; and follow-up referrals, if appropriate, with a primary care provider or other referred treatment resource). We define our primary patient outcome as the percent of adolescents who screened positive for AOD use and who reported linkage to discussions about AOD with a health care provider within 30 days of discharge.

#### Exploratory aim: measure of integration of opioid counseling

We examine the integration of prescription pain reliever counseling in two ways. First, we examine rates of documented opioid counseling in the EHR. Second, the Adolescent Perception of SBIRT Services Survey measure contains a question regarding if the adolescent received counseling/guidance about use of their prescription pain reliever medication.

#### Predictors of implementation effectiveness

During the initial administered staff survey to the three study leaders at each site, we will collect data on organizational level variables that might be associated with implementation effectiveness, which will control for as covariates in the model, e.g., pediatric trauma service volume, number of staff. In addition, we will track data on SBIRT knowledge and attitudes and staff engagement from utilization rates of the various training elements (i.e., percent of staff who attended the didactics, percent of staff who attended each webinar, etc.).

### Statistical methods and analysis

The outcome variable, fidelity to the SBIRT algorithm, will be summarized as the proportion of correctly complete versus incomplete or incorrectly completed SBIRT (i.e. complete = screened and appropriate SBIRT algorithm applied versus either not screened, or screened but not all appropriate elements of SBIRT algorithm applied). We will also examine the proportion of each SBIRT element appropriately applied. We will use a hierarchical modeling approach, to reflect the clustering of data within each pediatric trauma site, to model the longitudinal changes in SBIRT implementation. In the stepped wedge design, the implementation phases are not equally distributed across time, with more sites in the SSL preparation phase early in the study and more SSL sustainment phases later in the study. Potential temporal from implementation effects [26] will be disentangled in this study design. Time, implementation stage (SSL or no SSL), and the interaction of these variables will be modeled as fixed effects to determine if the change in SBIRT performance when a cluster changed from pre- to implementation status is consistent across the study. After accounting for the random effects of intracluster and site variability, we will determine the aggregate effects of implementation stage on SBIRT adherence.

Using the approach to mediational modeling proposed by Bauer et al. [27], which draws on the change in strength of the primary predictor variable (change from pre to SSL implementation step), when mediators are added to the multilevel model, we will test the effect of organizational readiness (the change from pre to implementation aggregated across sites), on the mixed effects model described above. We will compare the estimates for the effects of change in implementation stage when the mediator (average difference score pre and post-SSL implementation across each site) is added to the model.

Data gathered, from surveys with consented adolescent trauma patients 30 day after discharge, on linkage to care and continued AOD discussions after discharge, will be used as the outcome variable in this analysis. As with the hierarchical mixed effects model described above, we will include the nesting effects of site and cluster in this model, to account for variability in patient linkage that may be explained by both site and cluster variability. The change in report of 30-day linkage with appropriate discharge care will be modeled across time and implementation step, with the interaction effect described above. After adjusting for time, we anticipate that in aggregate there will be significantly greater proportion of patients who report post-discharge linkage to care as sites transition to SSL implementation.

There will be two sources of data for the outcome of counseling on use of prescription pain relievers. The patient interview at 30 days after hospital discharge and the medical record documention by the social worker who conducts the BI will be used. The initial analyses will present the percentage of medical record documentations of pain medication brief advice given by the social worker the report of this experience occurring given by the patient. This concordance rate will inform us not only of agreement between the two sources but also if there is a change in concordance when there is a site progression to SSL implementation. We will also model the change in rates of documentation or report of pain medication discussion using the general modeling approach previously described.

### Sample size estimates

The calculation of sample size and effective statistical power to meet the outcomes associated with our primary aim are based on the recommendations for stepped wedge designs [28, 29]. We conservatively estimate that there will be a 20% increase in SBIRT activities aggregated across the sites. Our sample size calculation is based on our smallest anticipated effects size; detecting an increase in documented referral to treatment following a positive alcohol screen (Cohen’s H = 0.28; *N* = 400 for an individual randomized trial). Using the formula provided by Baio et al. [28], assuming that the outcome variables are count data, and that the intracluster correlation is 0.20 (estimated from a prior study [8]), we would need a minimum of ten clusters, with six time points of data analyses to achieve a minimum of 80% power to detect a 20% change in the proportion of SBIRT activities completed with fidelity (α < 0.05, one-tail). We are requiring each site to review 30 medical records per wedge (total = 1800 across all sites and steps). We anticipate that a minimum of 25% of all patients screened will be positive for AOD use (based on national estimates from CDC’s Youth Risk Behavior Survey [30]), which would provide us with 450 AOD positive patients to test the RT component of our primary aim.

## Discussion

This protocol proposes a unique research opportunity to examine whether a comprehensive implementation strategy can improve the fidelity of SBIRT delivery across a national cohort of pediatric trauma centers. In addition to testing *whether* implementation strategies work, it is of paramount importance to examine *why* they work. Over the past decade, several conceptual models and frameworks have been developed for implementation research, each of which identifies constructs that are believed to help explain why implementation strategies are effective [31, 32]. However, a major limitation of implementation research to date has been a lack of studies empirically testing the extent to which these constructs explain (i.e., mediate) the effect of implementation strategy on implementation effectiveness. We aim to address this significant gap by testing the extent to which the effects of the SSL on implementation effectiveness are mediated by ORIC. ORIC is a multi-level construct that captures “the extent to which organizational members are psychologically and behaviorally prepared to implement organizational change [33].” When ORIC is high, members of an organization are expected to be more likely to initiate change, demonstrate persistence, and engage in cooperative behavior. This is expected to directly influence implementation effectiveness, which in turn directly influences the clinical effectiveness of the intervention. We expect that the SSL, which was designed to address barriers to change at the organizational level, will increase ORIC. We further expect that change in ORIC will directly predict implementation effectiveness (i.e., the extent to which staff deliver SBIRT with fidelity), which will in turn directly predict clinical effectiveness (i.e., the extent to which adolescents are linked to care following discharge from the pediatric trauma center). Finally, we expect that the effect of the SSL on implementation effectiveness will be explained (i.e., mediated) by ORIC.

The scientific significance of the research lies in its ability to advance implementation science by (a) testing whether the SSL implementation strategy can improve the fidelity of SBIRT delivery, (b) testing whether ORIC mediates the effect of the SSL on implementation effectiveness, and (c) utilizing ten pediatric trauma centers nationwide which increases the external validity and generalizability of study findings. Finally, its clinical significance for health care providers comes from advancing knowledge of how to improve compliance with ACS policy and how to improve clinical care of injured adolescents.

## Abbreviations

AAP: American Academy of Pediatrics
ACS: American College of Surgeons
ACS-COT: American College of Surgeons Committee on Trauma
AOD: Alcohol and other drug
ATTC: Addiction Technology Transfer Center
BAC: Blood alcohol level
BI: Brief intervention
BMI: Brief motivational interview
EHR: Electronic health record
IAMSBIRT: Implementing Alcohol Misuse Screening, Brief Intervention and Referral to Treatment
IRB: Institutional Review Board
LAR: Legal authorized representative
MI: Motivational interview
ORIC: Organizational readiness for change
PCP: Primary Care Provider
QI: Quality improvement
RT: Referral to treatment
S2BI: Screening to Brief Intervention Tool
SBIRT: Screening, Brief Intervention and Referral to Treatment
SSL: Science to Service Library
SUD: Substance use disorder
UDS: Urine drug screen
